# Update Review and Clinical Presentation in Congenital Insensitivity to Pain and Anhidrosis

**DOI:** 10.1155/2015/589852

**Published:** 2015-10-22

**Authors:** L. M. Pérez-López, M. Cabrera-González, D. Gutiérrez-de la Iglesia, S. Ricart, G. Knörr-Giménez

**Affiliations:** ^1^Pediatric Orthopaedic Surgery Department, Sant Joan de Déu Children's Hospital, University of Barcelona, Barcelona, Spain; ^2^Pediatric Rheumatology Department, Sant Joan de Déu Children's Hospital, University of Barcelona, Barcelona, Spain

## Abstract

*Introduction*. Congenital insensitivity to pain and anhidrosis (CIPA) or hereditary sensory and autonomic neuropathy type IV is an extremely rare syndrome. Three clinical findings define the syndrome: insensitivity to pain, impossibility to sweat, and mental retardation. This pathology is caused by a genetic mutation in the NTRK1 gene, which encodes a tyrosine receptor (TrkA) for nerve growth factor (NGF). *Methods*. The consultation of a child female in our center with CIPA and a tibia fracture in pseudoarthrosis encouraged us to carefully review literature and examine the therapeutic possibilities. A thorough review of literature published in Pubmed was done about CIPA and other connected medical issues mentioned in the paper. *Conclusions*. The therapeutic approach of CIPA remains unclear. The preventive approach remains the only possible treatment of CIPA. We propose two new important concepts in the therapeutic approach for these patients: (1) early surgical treatment for long bone fractures to prevent pseudoarthrosis and to allow early weight bearing, decreasing the risk of further osteopenia, and (2) bisphosphonates to avoid the progression of osteopenia and to reduce the number of consecutive fractures.

## 1. Introduction

Congenital insensitivity to pain and anhidrosis (CIPA), also known as hereditary sensory and autonomic neuropathy type IV, is an extremely rare syndrome. The first reference to a similar pathology was mentioned by Dearborn in the early 1900s [1], and it was published in 1963 by Swanson [2]. Three clinical findings define the syndrome: insensitivity to pain, inability to sweat, and mental retardation [3, 4]. Only a few hundreds of cases of CIPA have been recently published worldwide [5, 6]. This condition occurs with an incidence of 1 in 125 million newborns [7].

The pathogenesis of CIPA is characterized by a genetic loss-of-function mutation of the NTKR1 gene (locus 1q 21-22) [8, 9]. Multiple new mutations have been progressively described [10–16]. NTRK1 mutations imply an alteration in TrkA, a NGF receptor. NGF is involved in surveillance of nociceptive sensory neurons and sympathetic autonomic neurons and collaborates in the activation and homeostasis of other cellular types so that a NTRK1 mutation will cause deficient development of [17–20]

1. the afferent somatic sensory system for pain and temperature, located in the dorsal root ganglion sensory neurons,
2. the autonomic sympathetic neuronal system, which implies loss of the innervation of eccrine sweat glands by sympathetic neurons,
3. the central nervous system,
4. the bidirectional communication between the immune system and the nervous system (NGF has a relevant role in the signal pathway of B lymphocytes through three processes: Trk A phosphorylation, cytoskeleton assemblage, and MAP kinase activation).

The molecular alteration in the function of NGF in turn also alters the normal process of fracture consolidation [21]. Normal osteoblast/osteoprogenitor differentiation and proliferation are hindered, tending to result in fibroblast differentiation of multipotent stromal mesenchymal cells and periosteal cells.

Bone metabolism is also affected by the lack of nociceptive fibers, present not only in the skin but also in the skeletal system [22]. Due to the trophic role that nociceptive fibers may play in the skeletal system, bone fractures are very common [23].

## 2. Case Presentation

Medical record and radiographic data of the present case were reviewed and reported in a study approved by the department of documentation of our hospital. The patient's parents also gave their consent. A thorough review of the PubMed literature on CIPA and associated medical conditions mentioned in this paper was performed (Table 1). This case report is an illustrative example of a patient affected by CIPA.

We present a case involving a seven-year-old, female child of Spanish nationality. She had been evaluated in another center for episodes of recurrent fever. After a long diagnostic process including a pertinent genetic study which detected two mutations in the NTRK1 gene responsible for CIPA, she was diagnosed with the syndrome [8]. Her parents were healthy, and no consanguinity was present.

**Figure 1:**
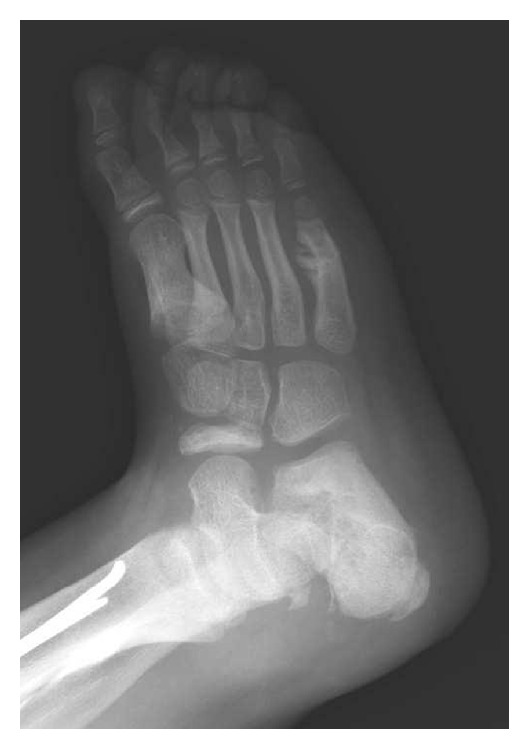
Navicular avascular necrosis and fifth metatarsal fracture in the right foot with hypertrophic bone callus.

**Figure 2:**
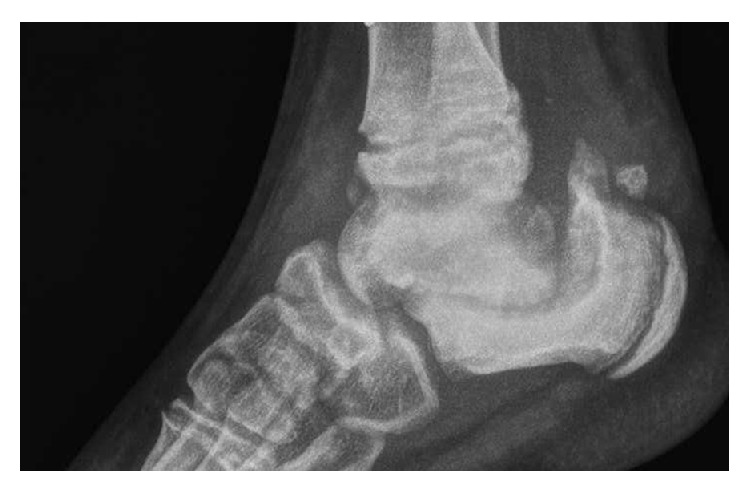
Demineralized bones and generalized osseous destruction.

**Figure 3:**
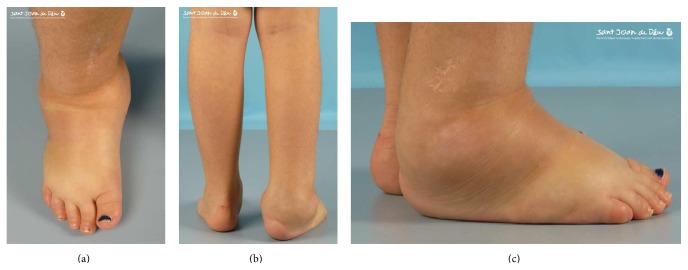
Lower limb edema.

Clinical exploration revealed absence of a pain response, recurrent episodes of fever, sweating deregulation, mental retardation, cutaneous autolesions, fracture without consolidation, avascular necrosis (Figure 1), demineralized bones, generalized osseous destruction (Figure 2), warm and dry skin with thickening of the soles and palms, and lower limb edema (Figures 3(a), 3(b), and 3(c)) [5, 24–26].

**Figure 4:**
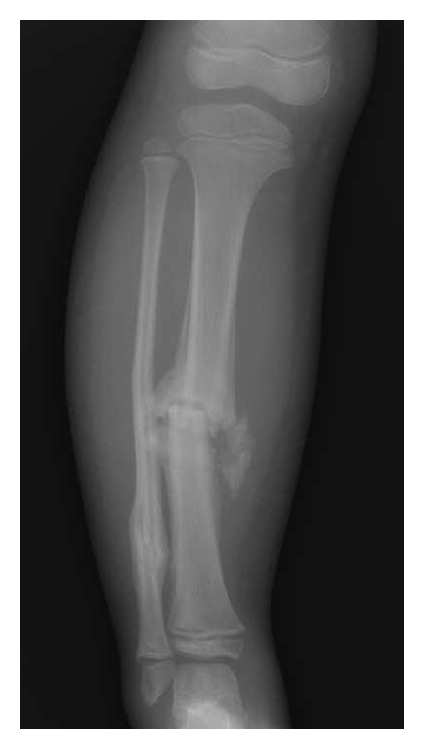
Hypertrophic bone callus.

**Figure 5:**
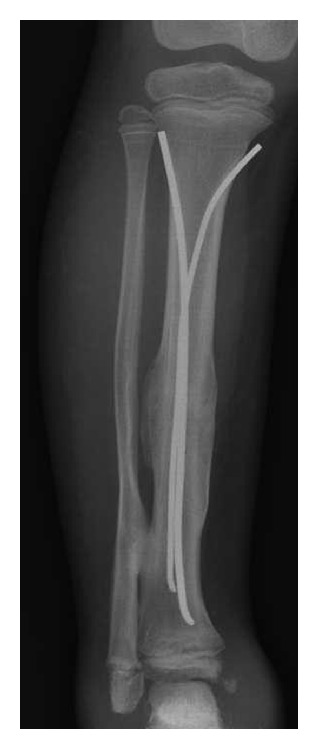
Complete radiological consolidation of the tibia fracture was achieved five months after the surgery.

The patient was referred to our center four months after fracture of the middle shaft of the right tibia. Radiologic signs of hypertrophic pseudoarthrosis were present (Figure 4). An elastic intramedullary nailing was carried out [27]. Complete radiological consolidation of the fracture was achieved five months after the surgery (Figure 5).

**Figure 6:**
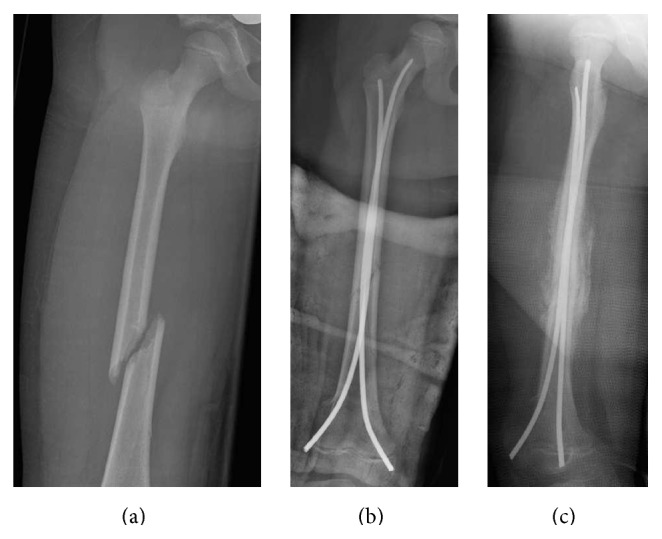
Right femoral middle shaft fracture that was surgically treated with good results. Hypertrophic bone callus associated.

In the following months, several fractures occurred, including a fifth metatarsal fracture in the right foot (Figure 1) and a fourth metatarsal fracture in the left foot, a right femoral middle shaft fracture that was surgically treated with good results (Figures 6(a), 6(b), and 6(c)), and an epiphysiolysis at the distal shaft of the right tibia. In CIPA, due to the alteration of the bone fracture metabolism, hypertrophic bone callus (Figures 1, 4, and 6(c)) and pseudoarthrosis (Figure 4) are very common. In the present patient, bone consolidation was only achieved when a surgical technique was applied.

During this period of time with recurrent fractures, treatment with bisphosphonates was started. A dose of 1 mg/Kg/day during 3 consecutive days of intravenous pamidronate was administered every four months, for one year. We obtained good results in preventing new fractures at upper and lower limbs, skull, and spine bones at 5 years of follow-up. No adverse effects were seen regarding pamidronate infusion or during the follow-up.

At 5 years of follow-up, patient has progressed.

## 3. Discussion

CIPA is an autosomal recessive disorder [8]. Some cases of consanguinity have been described among affected patients [7, 28]. Apart from the already well-defined genetic transmission of CIPA, there is an infrequent non-Mendelian inheritance characterized by uniparental disomy of chromosome 1 [10]. It is described by the transmission of an autosomal recessive pathology from only one affected parent. The molecular genetic analysis of the presented patient detected two heterozygous mutations in the NTRK1 gene (c.2205+1G>T in intron 16 and c.360-45C>A in intron 3), found also in her mother, suggesting then uniparental disomy.

The therapeutic approach to CIPA is still evolving and remains controversial [7]. There is no definitive agreement regarding its management, and therapeutic options are restricted to treatment of symptoms and protection from self-mutilation, fractures, and wound infections, which may lead to amputation. Such limited treatment options imply potentially catastrophic consequences of the natural pathologic evolution of the disease. Fractures associated with CIPA may be devastating and deeply affect the patient's functionality. Surgical treatment provides stability to the focal point of the fracture, helping to provide definitive consolidation. Moreover, immobilization contributes to accelerated bone demineralization. Surgical fracture repair allows for early weight bearing, diminishing the risk of further osteopenia, which is also usually present in these patients as a part of their associated neurogenic arthropathy (Figure 3) [21].

For all of these reasons, we recommend early surgical treatment of fractures. It allows for more rapid functional recovery, reducing the risk of accelerated osteopenia due to immobilization.

The use of bisphosphonates in patients affected byCIPA had never been mentioned before in literature. Due to our previous good experience with pamidronate in treating osteoporotic fractures for disuse in children with different medical conditions [29, 30], we made a therapeutic approach with pamidronate as a compassionate use in this child. We obtained good results in preventing new fractures.

These two therapeutic observations might be relevant in the absence of specific treatment for CIPA. However, we may not forget that further studies addressing CIPA management are needed to provide more rigorous and scientific conclusions.

CIPA may present various signs and symptoms that can be misleading. The differential diagnoses of this pathology include radicular hereditary sensory neuropathy (HSN I); hereditary sensory and autonomic neuropathy (HSN II); familial dysautonomia or Riley-Day syndrome (HSN III) [31]; congenital indifference to pain (HSN V) [32]; and Lesch-Nyhan syndrome. Corneal ulcers are also relatively frequent in patients with CIPA. A differential diagnosis of neurotrophic keratitis may be taken into consideration [33, 34]. Among all these diagnostic possibilities and according to Raspall-Chaure [29],

CIPA must be the first diagnostic hypothesis when assessing a patient with insensitivity to pain, anhidrosis, and self-mutilation.

According to literature, the first step in the diagnosis of CIPA syndrome is consideration of the clinical presentation based on the combination of three basic signs: insensitivity to pain, anhidrosis, and mental retardation [3, 4]. Other possible signs may be associated: impaired temperature sensation [5], facial alterations [6], mandibular osteolysis [7], dental caries [6], and premature tooth loss [6]; repetitive soft tissue and osseous infections of hematogenous origin [33], mainly caused by S. aureus [25]; self-mutilating behavior [7]; occasional microcephaly [5, 24]; urine and fecal incontinence [11]; growth disturbances; and heterotopic ossification [7, 35, 36].

Neurological laboratory tests may provide additional information. Short-latency somatosensory evoked potentials show marked prolongation of the central conduction time [19] and microneurography reveals abnormal activity of somatic A-delta and C fibers in the nerves of the skin [6, 37, 38]. A negative sympathetic skin response may also be helpful in the diagnosis due to the lack of sudomotor nerves in skin biopsy [38].

Pharmacologic tests that evaluate autonomic function are also useful. The Mecholyl test produces prompt pupillary miosis [24], pain test results abnormal [6, 13, 24], there is an absence of a flare reaction to the histamine test [24] (although we may find some normal responses to subdermal histamine injection) [11], and the sweat test using pilocarpine reveals a disruption of sweat gland function. Histopathologic evaluation shows a hyperplastic epidermis with acanthosis and hyperkeratosis and a decreased amount of sweat and sebaceous glands [6].

Finally, molecular evaluation that reveals mutations of the NTKR1 gene provides a definitive diagnosis [19, 24].

About the anesthetic considerations [39, 40], although pain stimuli are absent, anxiety associated with surgical procedures may generate stress and consequent hemodynamic instability. It is necessary to minimize preoperative apprehension and anxiety with the use of sedatives. Also the autonomic response to surgery is inconsistent and erratic, which results in difficulty determining the necessary anesthetic doses in advance. Finally, temperature control is crucial. Malignant hyperthermia or hypothermia may be lethal.

NGF-TrkA pathway has a role in the morphogenesis of the endocrine pancreas, in insulin secretion* in vitro*, and in insulin secretion in response to glucose. Patients with CIPA present with alterations of the first phase of insulin secretion [41].

The similarities between CIPA and reflex sympathetic dystrophy are very interesting. Both are characterized by neurogenic inflammation, skin alterations with vasomotor disruption, and osteopenia.

Some authors have focused on establishing a specific treatment for complex regional pain syndrome by studying the role of receptor tyrosine kinase for NGF in patients with CIPA [29].

The high incidence of infections in patients with CIPA is also problematic. Skin and deep bone infections are the most common types, and* Staphylococcus aureus* is the most commonly involved pathogen.

Resistance to antibiotics is a frequently occurring limitation in the treatment of these patients [25].

Temperature deregulation may cause recurrent fever, which may lead to death if not recognized early.

Other complications such as trauma or soft tissue/bone infection may decrease condition of the survival rate, although all are treatable conditions if diagnosed in a timely manner [7].

The best therapeutic approach to patients with CIPA appears to be based on prophylactic measures such as braces for early weight bearing in nonsurgical fractures and accurate follow-up to avoid missing complications. We propose an unusual treatment challenge, with an early surgical treatment for long bone fractures and early use of bisphosphonates as follows.

Therapeutic proposals are as follows:

1. Surgical fracture repair to achieve an early functional recovery that avoids a final destructive situation.
2. Bisphosphonates use to manage osteoporosis.

Addressing the cause of CIPA as opposed to solely symptomatic treatment seems to be the optimal therapeutic approach. If CIPA results from loss-of-function mutations in the NTRK1 gene encoding TrkA, then molecular treatment involving a receptor tyrosine kinase for NGF would be the most effective therapeutic technique.

## Figures and Tables

**Table 1:** Thorough review of the PubMed literature on CIPA and associated medical conditions mentioned in this paper was performed.

References	Year of publication	Particularity of the observation and remarks for each reading
Dearborn [1]	1932	First reference, in literature, to a similar disease

Swanson [2]	1963	First reference, in literature, to CIPA

Nishida [3]	1951	Three clinical representative findings: insensitivity to pain, inability to sweat, and mental retardation
Tunçbilek et al. [4]	2005	

Rosemberg et al. [5] review	1994	Only 32 cases have been published worldwide
Gao et al. [6]	2013	Only some hundreds of cases have been published
		worldwide

Daneshjou et al. [7]	2012	Incidence 1 in 125 million newborns

Indo et al. [8]	1996	CIPA pathogenesis: genetic loss-of-function mutation of
Indo et al. [9]	1997	the NTKR1 gene (locus 1q 21-22). NTKR1 mutations
		imply an alteration in TrKA, A NGF receptor

Indo et al. [8]	1996	Autosomal recessive disorder
Indo [10]	2001	Not only autosomal recessive inheritance, but also uniparental disomy (non-Mendelian inheritance of autosomal recessive disease from a single carrier parent, as the exposed case)

Indo [10] review	2001	Novel mutation and polymorphism in the NTRK1 gene causing CIPA
Indo et al. [15]	2001	
Bonkowsky et al. [11]	2003	
Lin et al. [12]	2010	
Mardy et al. [16]	2001	
Miura et al. [13]	2000	
Weier et al. [14]	1995	
Bonkowsky et al. [11]	2003	

Indo [25] review	2002	A very profuse resume of clinical and genetic characteristics of CIPA

Indo [18]	2010	NGF receptor failure causes a deficient development of dorsal root neurons (pain and temperature sensory system) autonomic sympathetic neural system (eccrine sweat glands innervation) Central nervous system The signal pathway of B lymphocytes
Indo [19]	2012	
Tanaka et al. [20]	1990	
Schwarzkopf et al. [17] review	2005	
Indo [25]	2002	
Melamed et al. [21]	2004	

Grills and Schuijers [24]	1998	NGF function disruption also causes an altered process of fracture consolidation

Fruchtman et al.[26]	2013	Descriptive clinical presentation including morbidity
Yang et al. [27]	2013	conditions (some of these clinical facts are also present in the case reported)

Jarade et al. [35] review	2002	Ocular manifestations

Brandes and Stuth [39]	2006	Anaesthetic considerations
Oliveira et al. [40]	2009	

Abdulla et al. [33]	2014	Heterotopic ossification and callus formation following fractures, eventually Charcot's joint

Schreiber et al. [41]	2005	Insulin-related difficulties
